# A Multimode Microfiber Specklegram Biosensor for Measurement of CEACAM5 through AI Diagnosis

**DOI:** 10.3390/bios14010057

**Published:** 2024-01-22

**Authors:** Yuhui Liu, Weihao Lin, Fang Zhao, Yibin Liu, Junhui Sun, Jie Hu, Jialong Li, Jinna Chen, Xuming Zhang, Mang I. Vai, Perry Ping Shum, Liyang Shao

**Affiliations:** 1 Department of Electronic and Electrical Engineering, Southern University of Science and Technology, Shenzhen 518055, China; 12068026@mail.sustech.edu.cn (Y.L.); 11510630@mail.sustech.edu.cn (W.L.); 12031197@mail.sustech.edu.cn (F.Z.); 11811808@mail.sustech.edu.cn (Y.L.); 12233189@mail.sustech.edu.cn (J.S.); 12031313@mail.sustech.edu.cn (J.H.); 12131038@mail.sustech.edu.cn (J.L.); chenjn@sustech.edu.cn (J.C.); shenp@sustech.edu.cn (P.P.S.); 2 Department of Applied Physics, Hong Kong Polytechnic University, Hongkong 999077, China; xuming.zhang@polyu.edu.hk; 3 Department of Electrical and Computer Engineering, Faculty of Science and Technology, University of Macau, Macau 999078, China; fstmiv@umac.mo; 4 Peng Cheng Laboratory, Shenzhen 518055, China

**Keywords:** CEACAM5, biosensor, specklegram, tapered MMF, 2D-CNN, ZNCC

## Abstract

Carcinoembryonic antigen (CEACAM5), as a broad-spectrum tumor biomarker, plays a crucial role in analyzing the therapeutic efficacy and progression of cancer. Herein, we propose a novel biosensor based on specklegrams of tapered multimode fiber (MMF) and two-dimensional convolutional neural networks (2D-CNNs) for the detection of CEACAM5. The microfiber is modified with CEA antibodies to specifically recognize antigens. The biosensor utilizes the interference effect of tapered MMF to generate highly sensitive specklegrams in response to different CEACAM5 concentrations. A zero mean normalized cross-correlation (ZNCC) function is explored to calculate the image matching degree of the specklegrams. Profiting from the extremely high detection limit of the speckle sensor, variations in the specklegrams of antibody concentrations from 1 to 1000 ng/mL are measured in the experiment. The surface sensitivity of the biosensor is 0.0012 (ng/mL)^−1^ within a range of 1 to 50 ng/mL. Moreover, a 2D-CNN was introduced to solve the problem of nonlinear detection surface sensitivity variation in a large dynamic range, and in the search for image features to improve evaluation accuracy, achieving more accurate CEACAM5 monitoring, with a maximum detection error of 0.358%. The proposed fiber specklegram biosensing scheme is easy to implement and has great potential in analyzing the postoperative condition of patients.

## 1. Introduction

CEACAM5 is a glycoprotein involved in cell adhesion. It is a protein component produced during the embryonic period and present inside the human body. After birth, the inhibition of glycoprotein causes the antigen content to gradually subside, as the baby gradually grows into adulthood. Normally, CEACAM5 in the colon and blood of adults is kept at a low level. Therefore, when serum CEACAM5 levels are elevated, it means that the body may suffer from cancer, including colorectal, lung, and breast cancers. CEACAM5 is thereby widely used as a broad-spectrum tumor marker. The concentration of CEACAM5 in the blood can predict cancer and monitor the treatment, progression, and prognosis of cancer [1,2,3]. In recent years, with the maturation of optical fiber manufacturing processes and a reduction of cost, a large number of optical fiber biosensors have been designed and utilized [4,5,6]. Optical fiber sensors have advantages such as their small size, light weight, and immunity to electromagnetic interference, which are extraordinarily suitable for monitoring label-free tumor biomarkers [7,8]. Wei et al. designed a microfiber coupler based on serum pre-adsorption to monitor CEACAM5 in blood, with a detection limit of up to 34.6 fg/mL [9]. However, its detection range is relatively narrow, and the normalization classification of antigen concentration between normal individuals and patients is liable to cause inaccurate results. Guan et al. used a microfiber interferometer under the synergistic effect of metal nanospheres to monitor CEACAM5 in serum, raising the detection limit to the order of 10^–16^ M [10]. Alternatively, the nanosphere adsorption process is complex and time sensitive. Hu et al. designed a lasso-shaped fiber sensor based on a fiber ring laser cavity. The sensor successfully detected 10 ng/mL of CEACAM5 [11]. Nonetheless, the low responsiveness of the structure to the external environment leads to sluggishness.

Currently, optical fiber specklegram sensors have been widely considered. They are able to achieve parameter measurements using relatively simple and inexpensive devices while still maintaining the advantages of traditional fiber sensors [12,13,14,15]. Consequently, there is a certain research significance in expanding optical fiber sensing technology. Based on the principle of mode interference, a specklegram carries numerous spatial state data. The impact of external environmental changes on optical fiber can be obtained through the output of a specklegram [16], thus it is capable of being applied to refractive index (RI) sensing. Zhao et al. analyzed in detail the responsiveness of a specklegram sensor based on single mode fiber—coreless fiber MMF structures for RI detection. The proposed method provides a good foundation for the subsequent application of specklegrams in biological sensing detection [17]. Besides, Li et al. improved the resolution of pressure sensors to 0.001 MPa by combining a Fabry Perot interferometric cavity with optical fiber speckle patterns [18]. Chen et al. introduced microfluidic into the fiber optic speckle sensing system and detected single-stranded DNA on the order of 10^−16^ M in 60 min [19]. Gangadharan et al. systematically discussed the possibility of the nondestructive testing of hydrogels [20], which played a leading role in flexible biosensor detection. Additionally, Monika et al. proposed a deep learning model in IoT systems [21], which has a good demonstration effect in the field of sensor detection. Notwithstanding the above schemes, a new fiber speckle technology for biomarker monitoring continues to be an urgent requirement.

In this study, a novel optical fiber biosensor based on the specklegram of tapered MMF and 2D-CNN training has been experimentally demonstrated for the measurement of CEACAM5. The mode excitation and energy distribution of lasers alters when the RI of the surrounding environment changes, resulting in the transformation of the output specklegram. The CEACAM5 antibody is specifically modified on the surface of optical fibers, and different concentrations of antigen adsorption can cause changes in the RI response of the fiber surface, resulting in changes in the specklegram [22,23]. The experimental results show that the designed biosensor has a detection range of 1–1000 ng/mL, and the waist width of the tapered MMF equals 7.46 μm. A ZNCC function is explored to demonstrate the surface sensitivity of the system in the range of 1–50 ng/mL, and the linear surface sensitivity of the system is 0.0012 (ng/mL)^−1^. A 2D-CNN algorithm is applied to achieve linear mapping within the full dynamic range, and the maximum detection error is 0.358%. The designed biosensor is expected to enhance the accuracy and practicality of existing label-free fiber optic biological monitoring systems.

## 2. Working Principle and Experimental Setup

**Figure 1:**
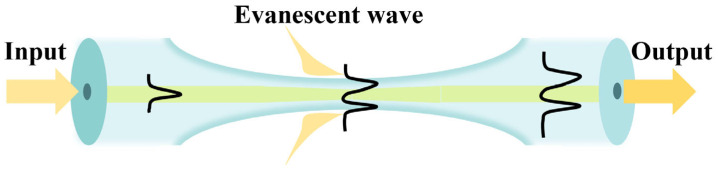
Schematic diagram based on the tapered MMF structure.

Fiber speckle is a gravelly pattern which appears on the output end surface of the fiber when the laser from a coherent source is transmitted through MMF. The field is created by interference between conduction modes in the fiber, as shown in Figure 1. Since the specklegram is sensitive to the guiding light conditions, the RI variation caused by the environmental fluctuations will lead to the alteration of output specklegrams. Thus, if the fiber is specifically modified, the pattern will be correlated with the external tumor marker concentration. The spatial intensity distribution of the output is a superposition of the vibration modes propagating throughout the fiber. MMF is worked as the optical transmission medium for analysis. The spatial distribution of amplitudes is as follows [24]:

(1) $$A\left(x,y\right)=\sum_{m=0}^{M-1}a_{m}\left(x,y\right)exp\left\{j\left[\varphi_{m}\left(x,y\right)\right]\right\}$$

where $A\left(x,y\right)$ is the spatial distribution on the output end face of MMF. M is the number of modes transmitted in the MMF. $a_{m}$ and $\varphi_{m}$ are the amplitude and phase distributions of the $m_{th}$ mode, respectively. The intensity $I\left(x,y\right)$ can be expressed as [24]

(2) $$I\left(x,y\right)=\sum_{m=0}^{M-1}\sum_{n=0}^{M-1}a_{m}\;a_{n}\left(x,y\right)exp\left\{j\left[\varphi_{m}-\varphi_{n}\right]\right\}$$

in which $a_{n}$ and $\varphi_{n}$ are the amplitude and phase distributions of the $n_{th}$ mode. If the fiber is subjected to the modulation of an external RI, the guided wave mode will be coupled, resulting in a variation in the mode power distribution. The expression for the power change of the $m_{th}$ mode is [24]

(3) $$∆P_{m}=\sum_{m=0}^{M-1}h_{mn}\left(p_{m}-p_{n}\right)$$

where, $p_{m}$ and $p_{n}$ are the initial power values of the $m_{th}$ and $n_{th}$ modes, and $h_{mn}$ is the coupling coefficient.

For fiber optic speckle demodulation technology, the selection of demodulation methods is crucial for establishing the relationship between specklegrams and external alterations. ZNCC is a commonly used algorithm for image matching processing. Compared with traditional cross-correlation analysis, ZNCC subtracts the reference specklegram and the average intensity of the current specklegram. By subtracting the local mean, the ZNCC algorithm compensates for the brightness changes of the obtained pattern, making it more robust to linear and uniform brightness conversions. The specific expression is [25]

(4) $$ZNCC=\frac{\iint \left(I_{0}-\bar{I_{0}}\right)\left(I-\bar{I}\right)dxdy}{{\left[\iint {\left(I_{0}-\bar{I_{0}}\right)}^{2}{\left(I-\bar{I}\right)}^{2}dxdy\right]}^{\frac{1}{2}}}$$

Here, $I_{0}$ and I represent the spatial intensity of the initial and detected specklegrams, respectively. $\bar{I_{0}}$ and $\bar{I}$ are the average intensities of the reference signal and measurement signal, respectively.

**Figure 2:**
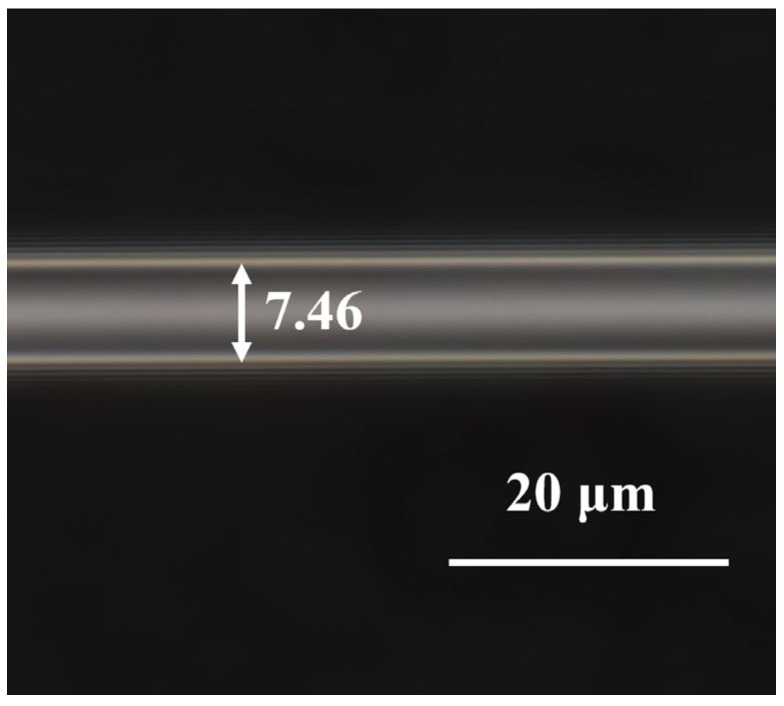
Micrograph of the tapered MMF.

Tapered MMF is made using a taper machine (Kepler, AFBT-8000LE-H0). The system is adjusted to standard mode and the tapering length set to 15,000 μm. Hydrogen and oxygen flame ignition is applied for tapering the fiber, resulting in MMF with a waist width of 7.46 μm, as shown in Figure 2.

**Figure 3:**
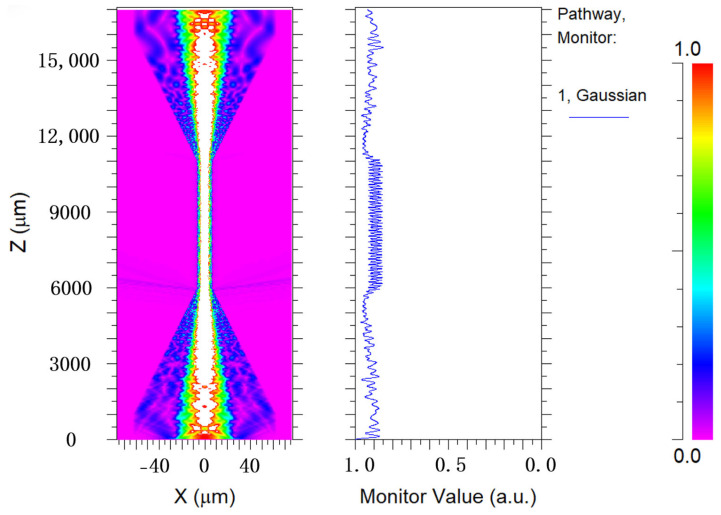
Simulation result of the mode field distribution for tapered MMF.

Based on the above manufacturing process, Rsoft software (version 2020; Synopsys, Mountain View, CA, USA) is employed to simulate the mode field distribution of the involved tapered MMF. The simulation results are shown in Figure 3; it is evident that various high-order modes are excited to generate interference phenomena in space when light passes through the tapered region. 

**Figure 4:**
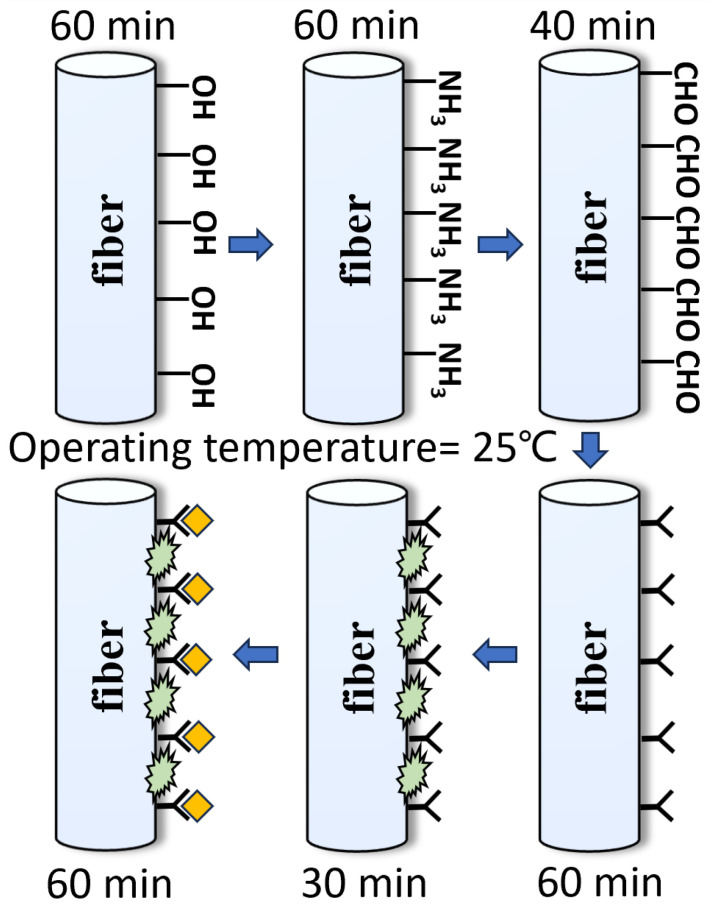
Preparation flowchart of the designed fiber biosensor.

With the aim of achieving the specific adsorption of antibodies on the fiber, the fiber was subjected to a series of pretreatments, as shown in Figure 4. Firstly, MMF was placed in a piranha solution for 1 h. The piranha solution was prepared by mixing 98% sulfuric acid and 30% hydrogen peroxide (3:1 by volume) to achieve hydroxylation of the optical fiber. After rinsing the soaked optical fiber in deionized water for 5 min, an additional three washes of ethanol solution are required to prevent hydrolysis of the silane solvent in the next step. The second step is to immerse the MMF in an ethanol solution containing 5% 3-aminopropyltriethoxysilane (APTES) and react for 1 h to realize the formation of amino groups on the surface. The third step is to submerse the MMF in a phosphate-buffered saline (PBS) solution containing 5% glutaraldehyde for 40 min. Glutaraldehyde adheres to the surface of the fiber through one of its amino-bonded aldehyde groups, leaving another free aldehyde group as a backup. The fourth step is to let the optical fiber soak in the PBS solution containing the CEACAM5 antibody with a concentration of 1 μg/mL for 60 min. A solution is prepared by diluting the antibody into PBS at pH 7.4 and immobilizing the CEACAM5 antibody using amino groups to bind to the aldehyde groups mentioned above, thereby forming a specific antibody on the surface of the optical fiber. Finally, the BSA solution is utilized to rinse the fiber for 30 min to occupy and block the unbound aldehyde groups on the surface to eliminate non-specific binding. Nine concentrations of CEACAM5 antigens, including 1, 5, 10, 20, 30, 50, 100, 500, and 1000 ng/mL were configured for this experiment. Antigens are prepared in PBS buffer to ensure biological activity and stability. Each concentration was tested for 1 h, and the groups were cleaned with 10 min of standard PBS buffer to ensure full binding of antigens and antibodies; each step was completed at room temperature.

**Figure 5:**
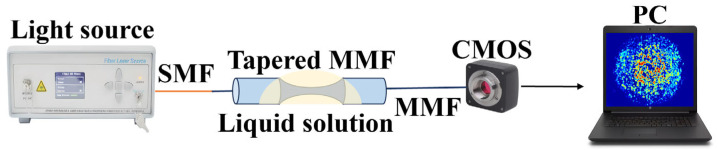
Configuration of the biosensor based on a tapered MMF structure.

Figure 5 shows the framework of the entire system. A fiber laser with a central wavelength of 1550 nm (MC Fiber Optics, MCNLFL-1550-S-S2-0-FA-T1) transmits energy into a MMF through a single mode fiber. When light is transmitted to the tapered MMF region, the path of transmission modes in the tapered fiber region is complex and diverse. The speckle is formed by the mixing and superposition of multiple modes. The evanescent field of the tapered fiber leaks into the external environment, hence the variation of RI caused by different antigens is able to affect the transmission of light within the fiber. The light carrying biological information maps one-dimensional information into two-dimensional specklegrams via a CMOS (CINOGY Technologies, CinCam CMOS-1201-IR) camera, and is recorded by a computer. The exposure time is 6 ms and pixel size is 5.3 μm × 5.3 μm. The distance between the fiber tip to the CMOS camera is 5 mm. The relevant parameters between fiber optic and CMOS cameras have a significant impact on the sensitivity of sensors, and that specific discussion is detailed in reference [26].

**Figure 6:**
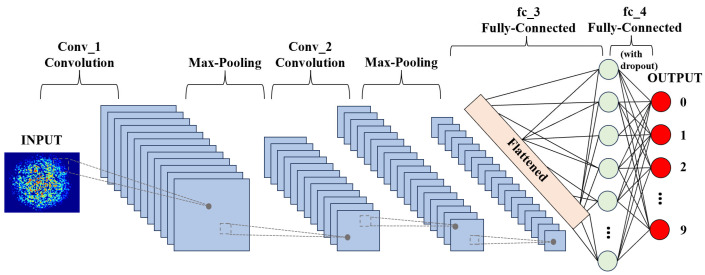
Scheme of biosensor recognition based on a 2D-CNN.

The application of 2D-CNN to achieve ultra-high precision prediction in a large dynamic range improves the practicability of biosensors. Its working principle is shown in Figure 6. Firstly, the input layer imports the collected image information into the system and pre-processes the original image data. In the next process, the convolutional layer extracts eigenvalues from the input image information; the pooling layer is sandwiched between successive convolution layers to compress the image and reduce overfitting. The compressed data is flattened so that it enters the fully connected layer, which unrolls all features and performs calculations to obtain probability values. That value is the probability that the input image is the expected concentration. The detailed execution information for the 2D-CNN is as follows: We first preprocessed the speckle image with a pixel size of 1280 × 1024 via normalization and converted the image to a gray level image. It was then normalized to the standard value of 0–1 and downsampled to 320 × 256; the hardware used for training was NVIDIA 3080 ti.

## 3. Results

**Figure 7:**
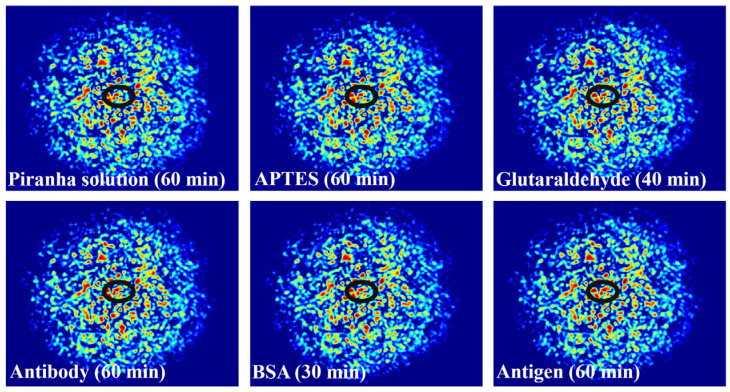
Specklegrams corresponding to the specific modification for each step.

Figure 7 shows the fiber specklegrams under different modifications. It must be acknowledged that the differences in specklegrams are extremely minute. However, within the marked black oval frame, clear dissimilarities in intensity and morphology can be seen, indirectly demonstrating the effectiveness of each functional processing and achieving specific modifications.

**Figure 8:**
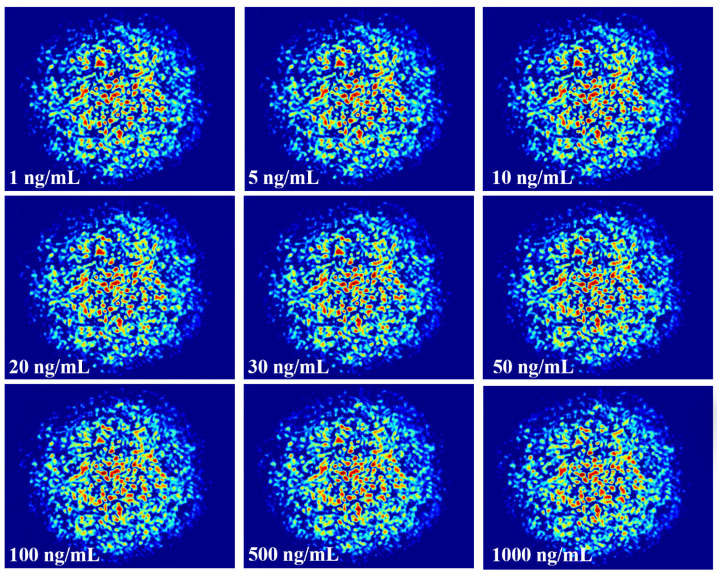
Specklegrams corresponding to different concentration of CEACAM5.

When light is transmitted in the fiber, due to the principle of total reflection, most of the energy is bound in the fiber core, and only a small part of the energy enters the envelope and the environment outside the fiber, that is, the evanescent field of the fiber. When the fiber pulls the lumbar spine thinner, more energy is spilt from the core, that is, the evanescent field strength increases, and the effect on the external environment is more intense. Because the structure of the tapered fiber has a cladding layer that is worn off, and its core is closer to the external environment than that of a conventional fiber, the evanescent wave at the cone region is more susceptible to a change of RI in the external environment, and can excite a large number of modes that cannot be supported by ordinary multimode fiber. The coupling between modes is stronger, and the spot pattern will be more random and contain more information. When the fiber is specifically modified, the specific antigen and antibody will be adsorbed on the fiber surface to change the RI, and the RI change will lead to the change of the speckle pattern, and the combination of antibodies and antigens with different concentrations will produce different RIs. The concentration of CEACAM5 can be specifically monitored by monitoring the speckle pattern of the refractive index change. The specklegrams generated by different concentrations of CEACAM5 are shown in Figure 8. It is difficult to visually observe distinctions. ZNCC normalizes the original data into data with zero mean and unit variance. Its basic principle is to use the range method, subtract the mean of each data value and then divide the range of the data so that the data value is 0 as the center, 1 as the upper and lower limits, and the data value is pulled to a standardized range. It can use specific values to calibrate the concentration, but also has the following advantages: 1. Noise elimination: the original data may contain a lot of noise; through zero-mean normalization you can greatly eliminate the noise and improve the accuracy of the model. 2. Enhance the comparability of the model: after zero-mean normalization, the data features will have stronger comparability, so the model can be better compared. 3. Optimize data processing: zero-mean normalization can effectively transform data features into a consistent format, which can better process data.

The correlation method of speckle image analysis has been widely used in vibration and temperature measurement as an effective means of optical measurement. This method is essentially a full-field deformation measurement technology based on digital image processing and numerical calculation. Its basic principle is to select a square image subregion around the pixel of interest in the source image and to obtain the displacement vector of the center point of the image subregion by tracking the position of the image subregion in the target image. The displacement field in the region of interest can be obtained by the same correlation calculation for multiple pixels in the region of interest in the reference image. In the actual analysis and calculation, in order to obtain accurate and reliable matching results, the selected image subregion should be large enough to contain sufficient gray change information, so as to ensure that the image subregion can be uniquely recognized in the deformed image. This requires that the surface of the measured object be covered with a speckle pattern, which acts as a deformation information carrier along with the specimen surface during deformation. Although speckle fields can be made on the surface of the tested sample by different methods, the gray distribution of speckle fields made by different methods or different personnel is very different, which leads to the histogram distribution, average gray level, image contrast, and other statistical parameters of the actual speckle map being completely different. Therefore, how to evaluate the speckle pattern as a carrier of deformation information is undoubtedly an important problem. Moreover, an effective speckle quality evaluation parameter will provide theoretical guidance for speckle production on the specimen surface, which is undoubtedly beneficial to improve the measurement accuracy of digital image processing-related methods. Strictly speaking, speckle is a randomly distributed grayscale map, and its correlation with the original pattern needs to be judged by dimensionality reduction, such as the ZNCC algorithm cited in this paper.

**Figure 9:**
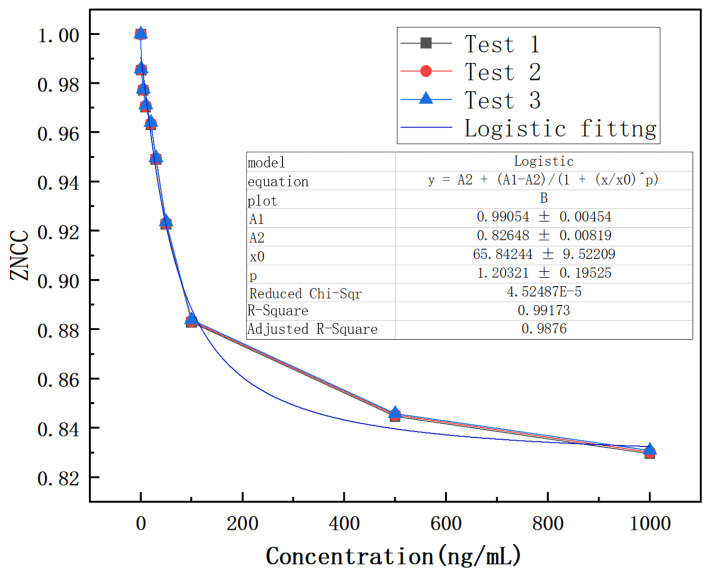
Logistic fitting of antigens over the concentration range of 1–1000 ng/mL.

**Figure 10:**
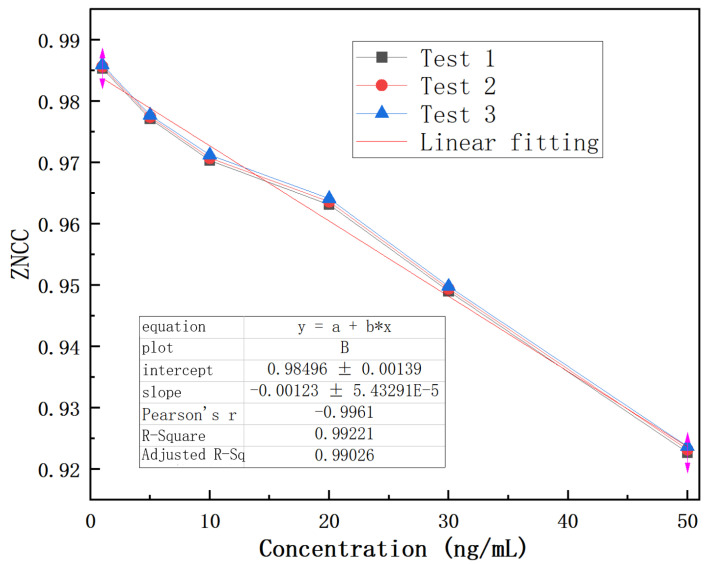
Linear fitting of antigens over the concentration range of 1–50 ng/mL.

Therefore, the ZNCC function is explored to analyze the results, as shown in Figure 9. The experimental result indicates that the designed biosensor has a response to CEACMA5 antigens ranging from 1 ng/mL to 1000 ng/mL and exhibits a nonlinear logistic regression fitting. A linear surface sensitivity response can be observed between 1 ng/mL and 50 ng/mL, as shown in Figure 10. The surface sensitivity of the sensor is 0.0012 (ng/mL)^−1^. We have experimentally proven that the detection limit of the sensor reaches 1 ng/mL. It must be emphasized that the experimental conditions must be stable, without any interference from external environmental factors, otherwise it will have a significant impact on the experimental result.

The poor stability of biosensing elements remains the main limiting factor for their widespread application. There are currently multiple solutions, such as enhancing the stability of biological components through molecular evolution or protein engineering methods; using extreme environmental organisms as biosensing sensitive elements since the cell elements are usually stable; adding stabilizers and protectants during the storage period of biosensitive components to extend their lifespan.

The self-compensation of sensing components is very important, and a standard speckle pattern can be set to quantitatively calibrate by subtracting it from the sensor’s speckle pattern to compensate for this problem.

**Figure 11:**
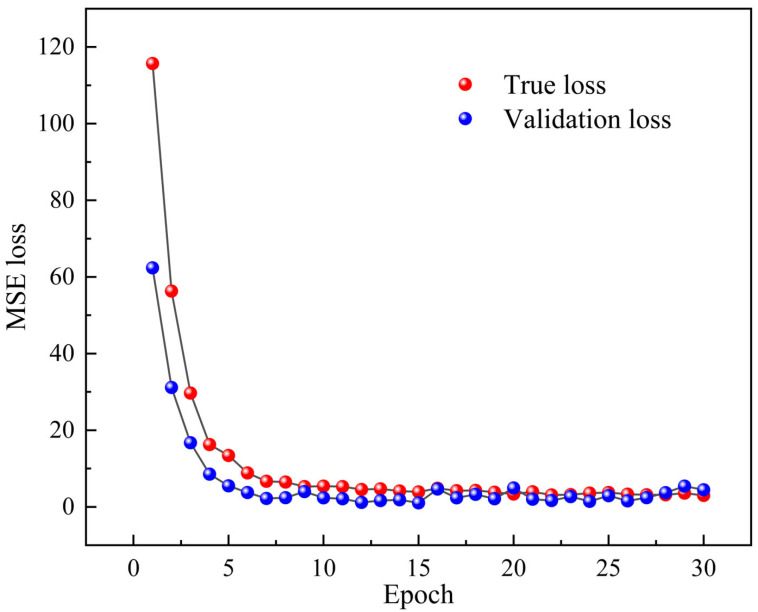
Learning curve based on 2D-CNN architecture.

The 2D-CNN model is operated to learn, train, and analyze the correlation between the designed fiber specklegram and the concentration of CEACAM5. Figure 11 demonstrates the learning curve, specifically represented as the relationship between loss and epoch. From the figure, it can be found that after 16 epochs, the function converges, proving that there are significant deviations between solutions of different concentrations. Besides, the model has the ability to judge the specific specklegrams corresponding to precise antigens. More importantly, the model can demonstrate excellent classification accuracy in both the training and validation sets, verifying the excellent generalization ability of this framework.

**Figure 12:**
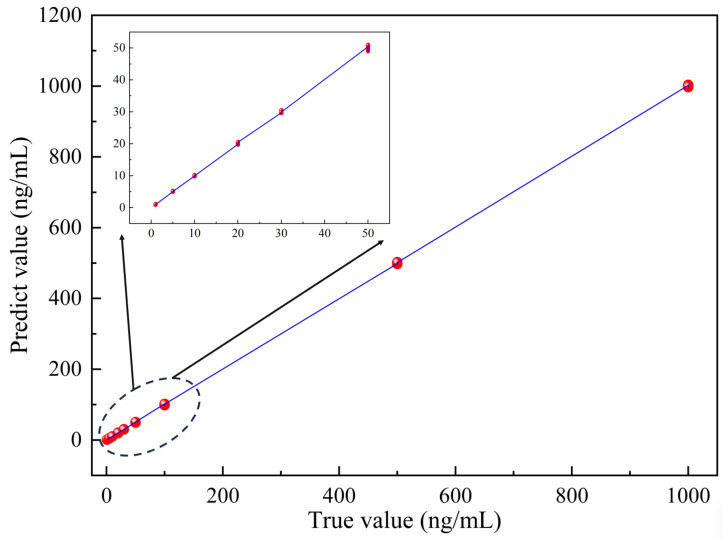
Output value between true concentration and predicted concentration.

A total of 900 specklegrams with 9 concentration gradients were collected in the experiment, of which 450, 225, and 225 were used for training, validation, and testing, respectively. The experimental results are shown in Figure 12. The predicted results maintain good consistency with the actual values, with a maximum error of only 0.358%; the slope equals 1.00008, demonstrating its outstanding predictive ability.

## 4. Conclusions

A novel optical fiber biosensor is proposed for measurement of CEACAM5 based on specklegrams and 2D-CNN training. The evanescent wave of a tapered MMF with a waist width equal to 7.46 μm is more susceptible to the surrounding RI, achieving a surface sensitivity of 0.0012 (ng/mL)^−1^ in the range of 1 ng/mL to 50 ng/mL. The specific adsorption of CEACAM5 antigens leads to a change in RI, resulting in changes in specklegrams. Moreover, the designed biosensor responds to antigens ranging from 1 ng/mL to 1000 ng/mL and can be fitted via a logistic function. In addition, a 2D-CNN is employed to analyze the input specklegrams and predict the practical volume fraction of CEACAM5. The loss function converged in the seven epochs, proving that machine learning is an effective method to analyze and predict concentration, with a maximum analysis error of 0.358%. The designed sensing system provides a new approach for fiber optic biosensing. More importantly, the sensor has the advantage of a high detection limit, which can have broad prospects for application in label-free tumor marker monitoring.

## Data Availability

Data are contained within the article.
